# Perception and Knowledge of Portuguese Veterinarians about the Zoonotic Transmission of *Helicobacter pylori* and *Helicobacter suis*: The Need for One Health Intervention

**DOI:** 10.3390/ijerph192215087

**Published:** 2022-11-16

**Authors:** Francisco Cortez Nunes, Silvia Teixeira, Rui Leandro Maia, Irina Amorim, Teresa Letra Mateus

**Affiliations:** 1 School of Medicine and Biomedical Sciences (ICBAS), University of Porto, 4050-313 Porto, Portugal; 2 Institute for Research and Innovation in Health (i3S), University of Porto, 4200-135 Porto, Portugal; 3 Institute of Molecular Pathology and Immunology of the University of Porto (IPATIMUP), 4200-135 Porto, Portugal; 4 UFP Energy, Environment and Health Research Unit (FP-ENAS), Universidade Fernando Pessoa, Praça 9 de Abril, 349, 4249-004 Porto, Portugal; 5 CITCEM—Centro de Investigação Transdisciplinar «Cultura, Espaço e Memória», Faculdade de Letras da Universidade do Porto, Via Panorâmica s/n, 4150-564 Porto, Portugal; 6 CISAS—Centre for Research and Development in Agrifood Systems and Sustainability, Escola Superior Agrária, Instituto Politécnico de Viana do Castelo, 4900-347 Viana do Castelo, Portugal; 7 EpiUnit—Instituto de Saúde Pública da Universidade do Porto, Laboratory for Integrative and Translational Research in Population Health (ITR), Rua das Taipas, nº 135, 4050-091 Porto, Portugal; 8 Veterinary and Animal Research Centre (CECAV), UTAD, Associate Laboratory for Animal and Veterinary Sciences (AL4AnimalS) Quinta de Prados, 5000-801 Vila Real, Portugal

**Keywords:** awareness, NHPH, One Health, public health, occupational health, risk communication, zoonosis

## Abstract

*Helicobacter* species can colonize the gastrointestinal tract of both humans and animals, and are associated with gastrointestinal and extra-gastrointestinal diseases. Some studies indicate that animals, health professionals, and people in close contact with animals might be at higher risk for infection with gastric *Helicobacter* spp. Considering that veterinarians are professionals at risk for infection with zoonotic gastric Helicobacters and are also seen by many as health communicators concerning zoonoses, the aim of this study was to evaluate the Portuguese veterinarians’ perception and knowledge of *Helicobacter* spp. infection and its zoonotic risk/potential. Therefore, a structured questionnaire composed of 34 dichotomic, multiple-choice, rating scale, matrix, drop-down, and open-ended questions was developed and given to Portuguese veterinarians via an online platform from May 2021 to July 2021, and statistical analysis was used to obtain results. There was a total of 149 respondents, most of them (73.8%) being females. Evidently, Portuguese veterinarians have a limited perception regarding *Helicobacter* spp. infections. Of the respondents that “*have heard of Helicobacter*”, 17.6% do not know which animal species can be affected by it. Most of the companion animal veterinarians (76.2%) do not consider *Helicobacter* spp. infection a differential diagnosis when evaluating animals with gastritis. A significant percentage (37.2%) of the respondents that have “heard of *H. suis*” do not consider it a zoonotic bacterium. There is a need for education and sensitization of veterinarians regarding the potential zoonotic risk of *Helicobacter* spp. in order to elucidate these professionals to this One Health issue, as the number of reports of non-*Helicobacter pylori Helicobacter* in livestock, companion, and wild animals is increasing.

## 1. Introduction

*Helicobacter* species can colonize the gastrointestinal tract of both humans and animals and are characterized as Gram-negative, spiral-shaped motile bacteria associated with several diseases [1,2,3,4].

In 1994, the World Health Organization (WHO) classified *Helicobacter pylori* (*H. pylori*) in the first group of carcinogenic agents, and its eradication remains a public health concern worldwide [5].

In humans, *H. pylori* is the most common gastric pathogen, affecting more than half of the world’s population, playing a major role in the development of gastritis, gastroduodenal ulcers, gastric adenocarcinoma, mucosa-associated lymphoid tissue (MALT) lymphoma, and extra-digestive diseases [1,6,7,8].

Gastric non-*Helicobacter pylori Helicobacter* (NHPH) have been associated with a wide range of pathologies, from MALT lymphoma to extra-digestive diseases, and have been diagnosed in 0.2–6% of human gastric biopsies [2,4,9].

In humans, *Helicobacter suis* (*H. suis*), naturally hosted by pigs, is the most prevalent NHPH and has also been associated with a range of gastric and extra-digestive pathologies [1,4,10,11,12,13]. Recent reports reinforce that these infections most likely originate from pigs, emphasizing their zoonotic potential [12,13,14,15].

*H. pylori* infection is acquired predominantly during childhood and persists throughout life without treatment [5]. The route of transmission is not 100% known and the human stomach is considered the only known reservoir, despite having been identified in other species’ stomachs [5,14,16,17]. Human-to-human transmission may occur through different pathways (such as gastro-oral, oral-oral, and fecal-oral), but there are other routes also hypothesized to be foodborne transmissions as the organism has been identified in food and water [5,17,18]. Indeed, *H. pylori* has been detected in drinking water, seawater, vegetables, and foods of animal origin. *H. pylori* survives in complex foodstuffs such as milk, vegetables, and ready-to-eat foods [19] and it has been demonstrated to transmit between humans and animals [20]. These types of contaminations are hypothesized to be the cause of new infections [5]*. H. suis* DNA was detected on pork carcasses in slaughterhouses, and the bacterium can persist for up to 48 h in experimentally contaminated pork. Therefore, pork consumption may constitute a new route of *H. suis* transmission in humans [21]. *H. suis* has also been associated with gastric disease in animal health professionals, emphasizing its zoonotic importance [12]. Thus, as some studies indicate, animals, health professionals, and people in close contact with animals might be at a higher risk for infection [22].

Considering that veterinarians are professionals at risk for infection with zoonotic species of gastric Helicobacters, and are also seen by many as health communicators concerning zoonoses, the aim of this study was to evaluate the Portuguese veterinarians’ perception and knowledge of *Helicobacter* spp. infection and its zoonotic risk/potential.

## 2. Materials and Methods

### 2.1. Data Collection

Data collection was performed using a structured questionnaire, specifically developed for this study in Portuguese. The draft questionnaire was designed based on the bibliographic review and three focus groups with veterinarians. Then, it was tested before being released. The questionnaire includes information related to general demographic (date of birth, gender, region), contact with animals (specifically pigs and wild boars), and the context and duration of the contact. Information regarding food consumption, consumption of pig and wild boar meat or meat products, as well as the frequency of weekly consumption and food safety, was also assessed. Moreover, data regarding perception and knowledge of *Helicobacter* spp., *H. suis*, *H. pylori*, infection, and zoonotic risk was assessed. Data regarding the possible gastric disease of each participant presumably associated or not with *Helicobacter* spp. infection was also addressed. Finally, these data were collected regarding the participants’ interest in receiving further information about *Helicobacter* spp. infection in animals and humans and how they would like to receive any further information.

The following variables were considered for the present study: general demographic characteristics, contact with animals, nutritional habits, food safety habits, veterinarians’ knowledge of *Helicobacter* spp., perception of each individual gastric pathology, and receptiveness to obtain more information regarding the survey topic.

This process resulted in the final survey, composed of 34 dichotomic, multiple-choice, rating scale, matrix, drop-down, and open-ended questions.

### 2.2. Study Design and Participants

A cross-sectional study using data from an online survey based on the snowball sampling method was performed [23]. A virtual snowball sampling survey was disseminated, firstly, through closed social networking channels (namely, Facebook^®^, Menlo Park, CA, USA) and, secondly, through the researcher’s mailing lists to work colleagues and veterinarian friends. The inclusion criteria consisted of age ≥23, being a certified veterinarian, and being based in Portugal. Everyone who followed the link accepted to participate. Since the survey did not have information on where the participants were recruited (e.g., Facebook or mailing list), due to confidentiality, we cannot calculate the proportion of participants that come from each networking channel. For the present study, data was gathered from May 2021 to July 2021, corresponding to a convenience sample of 149 individuals.

### 2.3. Ethics Approval and Consent to Participate

Ethical approval was obtained from the i3S Animal Welfare and Ethics Review Body (ref.2021-4). According to the Ethical Principles for Medical Research involving human subjects expressed in the Declaration of Helsinki and the current national legislation, all participants were asked to give their informed consent. Since this was an online survey, participants had to select the option: “accept to participate in the study to proceed with the survey”. The questionnaire was confidential, and no data that allowed the identification of the participants was collected.

### 2.4. Statistical Analysis

These data were analyzed using IBM SPSS^®^ Statistics v27 (IBM, Armonk, NY, USA). Descriptive statistics were generated to verify the variables described above. Cross-tables and chi-square analyses were used to explore possible associations between nominal variables and Cramer’s V test, with an oscillation between 0 and 1, to assess the intensity of the relationship between variables. Statistical significance was set at *p* < 0.05.

## 3. Results

### 3.1. Sociodemographic Distribution

Of the 149 questionnaires were completed, 73.8% (110/149) of the respondents were female veterinarians and 26.2% (39/149) were male veterinarians. The mean age was 37.2 years-old and 71.1% (106/149) were companion animal veterinarians.

**Figure 1:**
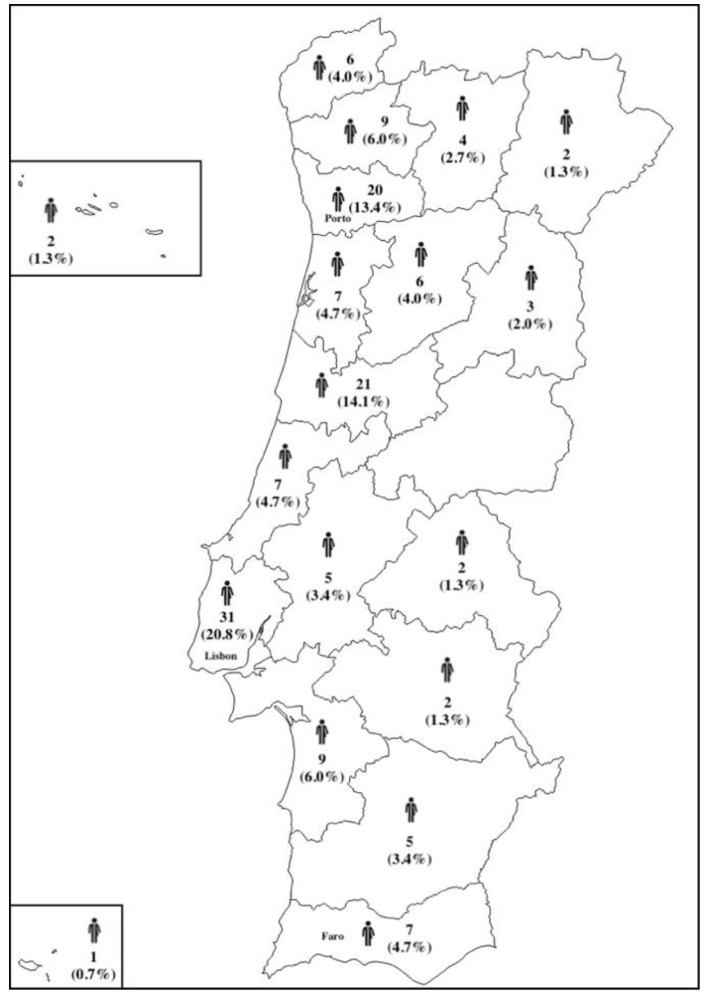
Distributions of the respondents enrolled in the questionnaire.

There were respondents from 17 of the 18 districts of Portugal with the greater percentages of respondents from Lisbon (20.8%), Coimbra (14.1%), and Porto (13.4%) in concordance with the most populated regions in Portugal (Figure 1).

### 3.2. Knowledge about Helicobacter spp. Infection

When the questionnaire asked “*have you heard of Helicobacter?*”, only 0.7% (1/149) chose “*no*” and 99.3% (148/149) chose “*yes*”. Of the respondents that chose “*yes*”, 97.3% (144/148) answered “*yes, I know what it is*” and 2.7% (4/148) “*yes, but I don’t know what it is*”.

Of the 148 respondents that “*have heard of Helicobacter*”, when asked the question “*have you heard of H. suis?*”, 47.0% replied “*no*”, 38.3% replied “*yes, and I know what it is*”, 13.4% replied “*yes, but I don’t know what it is*”, and 0.7% preferred not to answer. Regarding the question “*Have you heard of H. pylori?*”, 2.0% selected “*no*”, 92.6% selected “*yes, and I know what it is*”, and 4.7% selected “*yes, but I don’t know what it is*”.

From the 148 respondents that “*have heard of Helicobacter,*” 17.6% did not know which animal species could be affected by *Helicobacter*, and only 4.0% stated that they “*do not know*” clinical signs associated with *Helicobacter* spp. infection in animals or humans.

Of the 106 respondent veterinarians who practice in companion animal clinics, the majority never (22.6%) or rarely (50.0%) consider *Helicobacter* spp. infection as a differential diagnosis of gastritis in companion animals; on the other hand, 21.6% frequently and 3.8% always consider it.

Of those 106 companion animal veterinarians, 22.6% have already diagnosed gastritis associated with *Helicobacter* spp. infection while 54.7% never have. Within the 24 companion animal veterinarians that have diagnosed gastritis associated with *Helicobacter* spp. infection, 22.6% diagnosed it in dogs, 8.3% in cats, and 12.5% in other species.

Of the 149 respondents, 47.7% consider *Helicobacter* spp. a zoonotic bacteria, 13.4% do not consider it a zoonotic bacteria, and 38.9% “*do not know*”.

Although 71 veterinarians consider the zoonotic potential of *Helicobacter* spp., most (45.1%, 32/71) referred to an ambiguous answer when asked how the transmission between animals and humans occurs. Some indicated “*food contamination*” (21.1%, 15/71), “*feco-oral route*” (16.9%, 12/71), or “*oral-oral route*” (8.5%, 6/71), while 11.2% (8/71) stated that they “*do not know*”.

### 3.3. Contact with Pigs and Wild Boars and Consumption of Their Meat or Meat Products

Of the total respondents, 79.2% (118/149) had contact with pigs and/or wild boars: 31.4% (37/118) as livestock animal veterinarians, 23.7% (28/118) as companion animal veterinarians, 11.9% (14/118) as meat inspectors, 10.2% (12/118) when they were students, 1.7% (2/118) as hunters, 1.7% (2/118) as livestock farmers, and 19.5% (23/118) in other circumstances.

Of the 118 veterinarians who had contact with pigs and or wild boars, 52.5% have been in contact with pigs for less than 5 years and 47.5% for more than 5 years.

Regarding consumption of pig and wild boar meat or meat products: 89.9% (134/149) consumed pork at least once per week and 90.6% (135/149) consumed pork products, 38.9% (58/149) consumed wild boar meat at least once per week, and 18.1% (27/149) consumed wild boar meat products at least once per week.

### 3.4. Food Safety and Hygiene

More than one-fourth of the veterinarians (28.8%, 34/149) refer to the use of borehole water during food preparation or for ingestion.

Regarding the preparation of food at home, 98.7% (147/149) prepare food at home at least once per week.

Of the 147 respondents that prepare food at home, 97.9% (144/147) state that they “*have hygiene and safety measures during food preparation*”, 0.7% (1/147) state that do not have hygiene and safety measures during food preparation, and 1.4% (2/147) state they “*do not know if they have hygiene and safety measures during food preparation*”. When questioned about which type of hygiene and safety measures they practice: 81.3% (117/144) mentioned “*food washing*”, 45.1% (65/144) “*cooking*”, 27.8% (40/144) “*food separation to avoid cross-contamination*”, 10.4% (15/144) “*refrigeration”,* and 3.5% (5/144) “*hand washing”*.

### 3.5. Gastric Pathology among Veterinarians

Of the respondent veterinarians, 83.9% (125/149) admitted to having “*gastric pain*”, 69.1% (103/149) “*had gastritis*”, 77.2% (115/149) “*suffered from gastric reflux/heartburn*”, and 30.9% (46/149) did some type of gastric diagnostic test (Table 1). Finally, 21.5% (32/149) of the respondents are medicated for gastric disease.

A total of 22 (14.7%) of the 149 participants responded that they had gastric disease associated with *Helicobacter*. Of this number, 100% (22/22) claim to have been treated for *Helicobacter pylori* infection, with 50.0% (11/22) cured or eradicated, 18.2% (4/22) not cured or eradicated, and 31.8% (7/22) did not know if they got cured or if eradication was achieved.

### 3.6. Interest in Receiving Information on Helicobacter spp. Infections

Lastly, the respondents were questioned if they would like to receive more information about *Helicobacter* spp. infection in livestock animals, companion animals, or humans. Most (85.9%, 128/149) said “*yes*”, and 32.0% (41/149) responded that they would like to get it through the Portuguese Order of Veterinarians (*Ordem dos Médicos Veterinários*, OMV).

### 3.7. Statistical Associations between Variables

When applying statistical analysis to determine if there was any association between them, it was possible to conclude that there are some statistically significant associations (with green background in Table 2) whose strengths vary from weak (<0.1) to moderate (<0.3) considering the Cramer’s V and the degree of freedom =1.

## 4. Discussion

There are several species of gastric Helicobacters*,* with *H. pylori* being one of the most studied since humans remain the natural host. *H. pylori* is associated with gastric disease as it may cause gastritis, peptic ulcer disease, gastric carcinoma, and mucosa-associated lymphoid tissue (MALT) lymphoma. Besides this species, other NHPHs with zoonotic potential, such as *H. suis*, have been associated with gastric and neurologic pathologies in humans [2,4,10,24,25]. Considering the close contact between humans and animals and the late reports of *Helicobacter* spp. in humans [26], the late reports of *H. pylori* human-animal transmission and infection [20], or the detection of *H. pylori* DNA in other animal species [14,16], and also the description of human *H. suis* infection [1,27], including a report in a veterinarian [12], our goal was to investigate the awareness of veterinarians regarding *Helicobacter* species, such as *H. pylori* and *H. suis.*

However, there are two limitations that should be pointed out: one is the small sample size and the other is that the majority of the respondents were female veterinarians, so the generalizability of our findings is limited.

Most of the 149 questionnaire respondents were females (73.8%), as expected according to the 2018 European veterinary survey applied and published by the Federation of Veterinarians of Europe (FVE) [28]. Additionally, according to the OMV, on the 12th February 2020, there were 4071 (61%) female veterinarians and 2551 (39%) male veterinarians registered in Portugal. Therefore, when associating different variables with gender, there is expected to be a deviation towards the female gender.

Despite some of the NHPHs being considered zoonotic or with zoonotic potential [25,29], in this study 37.2% of the respondents that “*heard of H. suis*” do not consider it a zoonosis (*p* < 0.001; cv = 0.322). However, people directly affected by *H. pylori* infection seem to be aware of this hypothesis since 68.2% of the respondents that “*had gastric disease associated with Helicobacter*” (*p* = 0.037; cv = 0.171) and 68.2% that “*had treatment for Helicobacter infection*” (*p* = 0.029; cv = 0.219) consider it a zoonosis.

Interestingly, 59.0% of the respondents between the ages of 25 and 35 years-old consider *Helicobacter* spp. infections a zoonosis, while only 39.8% of the respondents between the ages of 36 and 68 years-old do (*p* = 0.021; cv = 0.189). We hypothesize that the recent emergence of zoonoses globally [30] may have made younger professionals more aware of these diseases.

Some studies report the presence of *H. suis* on pig carcasses and retail meat which can pose a transmission route [21,31]. Intriguingly, 42.4% of the respondents to the questionnaire that claim “*to eat pork*” regularly also “*have gastric pain*” (*p* = 0.047; cv = 0.163). Although no direct association can be made, this result should be explored in further studies.

Regarding food safety and hygiene, despite 97.9% of the respondents that prepare food at home stating that they “*have hygiene and safety measures during food preparation*” (*p* < 0.01; cv = 0.398), only 3.5% mention “*hand washing*” as a food safety practice. Similar findings were reported in a study by Stratev et al. (2017) concluded that, despite the high level of awareness of food safety, these practices were low among veterinary medicine students in Bulgaria [32].

With our study we were able to assess that most of the respondents have limited perception and knowledge about the zoonotic/anthropozoonotic risks of *H. suis* and *H. pylori* infections. When questioned “*have you heard of Helicobacter*”, 99.3% chose“*yes*” with the majority of the respondents (97.3%) knowing *H. pylori* but only 51.7% acknowledging *H. suis*, and interestingly, 17.6% of respondents that “*have heard of Helicobacter*” did not know which animals’ species could be affected. Furthermore, 13.4% do not consider *Helicobacter* spp. a zoonotic bacteria and 38.9% “*do not know*”, even though 96.6% claimed that they “*have heard of Helicobacter and know what it is*” and 38.3% “*have heard of H. suis and know what it is*”.

It can also be hypothesized that *Helicobacter* spp. infections among animals could be underdiagnosed due to a lack of knowledge/awareness of veterinarians regarding this bacterial infection. In fact, 17.6% did not know the animal species that could be affected, and of the 106 veterinarians that practice at companion animals’ clinics, 72.6% never (22.6%) or rarely (50.0%) consider *Helicobacter* spp. infection as a differential diagnosis of gastritis in companion animals.

When assessing gastric pathology among the respondents, 59.4% of females responded positively to “*did you use to have gastric pain*” and 68.2% of females “*have had treatment for Helicobacter*” (*p* = 0.003; cv = 0.277), which follows the study published by Khoder et al. (2021) that reports a higher occurrence of *H. pylori* infections in females [33].

We also concluded that veterinarians with higher contact time (>5 years) with pigs (52.9%) also “*had had treatment for Helicobacter infection*” (*p* = 0.020; cv = 0.268), which again may indicate the occupational risk of these professionals as reported by Joosten et al. (2013) [12].

Remarkably, 86.5% of the respondents that “*have heard of Helicobacter spp*.” (*p* = 0.013; cv = 0.203), 85.7% that “*have not heard of H. suis*” (*p* = 0.045; cv = 0.204), 87.2% that “*have heard of H. pylori*” (*p* = 0.001; cv = 0.299), 100% that “*take medication for gastric pain*” (*p* = 0.010; cv = 0.212), 100% that “*have gastric problems associated with Helicobacter infection*” (*p* = 0.040; cv = 0.169), and 100% that “*had treatment for H. pylori infection*” (*p* = 0.023; cv = 0.226) would like to receive more information about this bacterial infection in livestock, companion animals, and humans. This may suggest that despite having some insights about the subject, they would like to get further information and enrich their knowledge.

On the other side, we should not neglect that a surprising percentage of veterinarians (14.1%) do not wish to receive additional information.

To the authors’ best knowledge there are no studies that assess the perception, awareness, and knowledge of veterinarians regarding *Helicobacter* spp. infections. This makes our results the first to be reported and challenging to compare since there are no similar studies among veterinarians that are up to date.

It is known that zoonotic diseases are a growing concern, amounting to approximately 60% of the existing human pathogens, and over 75% of those can be tracked to animals [34]. Sometimes, zoonotic diseases manifest in animals before they infect humans [35], so veterinarians possess specialty training in zoonoses and, thereafter, should be a great resource of information on this subject [36]. In addition, m according to Spear et al. (2015), people would be willing to consult with a veterinarian on the advice of their physician if they had a zoonotic disease [36]. Some studies also show that veterinarians should be active not only in controlling zoonotic diseases in animals but also in providing information for patients and physicians, and that physicians assign them the duty of educating populations about zoonoses [36,37,38]. This happens because physicians are uncomfortable with their knowledge about zoonoses [36,38]. Therefore, physicians consign veterinarians the duty of educating the public about zoonoses, yet neither of these experts communicate with the other [35].

Despite the results of our study being suggestive of limited knowledge and awareness of veterinarians regarding *Helicobacter* spp. infections, they continue to play an important role in the promotion of public health [35,39]. Pet owners are more likely to contact their veterinarian than their physician regarding information about zoonoses [40], along with the fact that they consider the role of veterinarians important as public health promoters [39]. So further training programs addressing vet communication skills should take into account the particular issues of emerging zoonoses, and veterinarians need to have proper knowledge so they can assess and explain the risks to their clients [41].

The medical and veterinary communities should collaborate closely in clinical, public health, and research contexts since zoonoses can infect both animals and humans [42].

Veterinarians are in the perfect position to give animal owners trustworthy information since they are more aware of the possible hazards of zoonotic diseases and how to minimize them.

There is a need for collaboration between animal, human, and environmental professionals in an objective One Health perspective due to the increase in emerging zoonotic diseases. Veterinarians seem to show greater awareness of the importance of cooperation activities and continuous cross-sectional formation than physicians [34].

Nonetheless, communication between veterinarians and physicians seems to be insufficient [37], so it is important to create awareness of this need and promote cooperation and communication since the One Health initiative aims to reduce this professional gap between veterinarians and physicians [43], and both parts can and should play a role on public health education.

## 5. Conclusions

There is a need for education and sensitization of veterinarians regarding the zoonotic risk of *Helicobacter* spp. in order to elucidate these professionals to this One Health issue since the number of reports of NHPH in livestock, companion, and wild animals are increasing.

The results of this study allowed us to conclude that communication and sensibilization regarding *Helicobacter* spp. infections in animals and its zoonotic potential should be done by veterinarians since there are 37.2% of them that heard of *H. suis* but do not consider it a zoonosis. A special focus should be implemented regarding veterinarians older than 35 years since only 39.8% of these consider *Helicobacter* spp. infections as potential zoonoses.

The veterinarian respondents demonstrated interest in receiving more information regarding *Helicobacter* spp. infections in production animals, companion animals, or humans which could, in fact, increase their awareness on this topic, and improve their role as clinicians and public health agents.

## Figures and Tables

**Table 1:** Assessment of veterinarians’ gastric pathology: questions and answers (numbers and percentages).

Question	N	Answer	%
Did you use to have gastric pain?	149	Never	16.1
		Rarely	61.7
		Frequently	20.8
		Always	1.3
Have you had gastritis?	149	Never	30.9
		Rarely	55.7
		Frequently	13.4
Do you suffer from gastric reflux/heartburn?	149	Never	22.8
		Rarely	54.4
		Frequently	20.8
		Always	2.0
Have you done any gastric diagnostic test?	149	Yes	69.1
		No	30.9
If yes, what diagnostic test was performed?	103	Endoscopy only	56.5
		Endoscopy with biopsy	19.6
		Abdominal echography	8.7
		Rapid urease test	6.5
		Abdominal radiographs	2.2
		Ambiguous answer	6.5
Do you take any medication for gastric problems?	149	No	78.5
		Yes	21.5
If yes, which?	32	Omeprazole	50.0
		Pantoprazole	31.3
		Sucralfate	31.3
		Esmoprazole	21.9
		Antibiotics	6.3
		Ranitidine	3.1

**Table 2:** Statistical association between variables.

Question		Gender	Age	Years of Contact with Pigs	Do You Eat Pig Meat?	Do You Use Borehole Water to Prepare Food?	Do You Prepare Food at Home?	Do You Consider *Helicobacter* spp. Infection a Zoonosis?	Would You Like to Receive More Information about *Helicobacter* spp. Infection in Production Animals, Companion Animals, or Humans?
	Variables	Male/Female	[25–35]/[36–68]	[0–5]/[>5]	Yes/No	Yes/No	Yes/No	Yes/No	Yes/No
Have heard of *Helicobacter* spp.?	Yes/No	*p* > 0.05	*p* > 0.05	*p* > 0.05	*p* > 0.05	*p* > 0.05	*p* > 0.05	*p* > 0.05	*p* = 0.013; cv = 0.203
Have heard of *H. suis*?	Yes/No	*p* > 0.05	*p* > 0.05	*p* > 0.05	*p* > 0.05	*p* > 0.05	*p* > 0.05	*p* < 0.001; cv = 0.322	*p* = 0.045; cv = 0.204
Have heard of *H. pylori*?	Yes/No	*p* > 0.05	*p* > 0.05	*p* > 0.05	*p* > 0.05	*p* > 0.05	*p* > 0.05	*p* > 0.05	*p* = 0.001; cv = 0.299
Do you consider *Helicobacter* spp. infection a zoonosis?	Yes/No	*p* > 0.05	*p* = 0.021; cv = 0.189	*p* > 0.05	*p* > 0.05	*p* > 0.05	*p* > 0.05	*p* > 0.05	*p* > 0.05
Did you use to have gastric pain?	Yes/No	*p* > 0.05	*p* > 0.05	*p* > 0.05	*p* = 0.047; cv = 0.163	*p* > 0.05	*p* > 0.05	*p* > 0.05	*p* > 0.05
Do you take any medications for gastric problems?	Yes/No	*p* = 0.036; cv = 0.172	*p* > 0.05	*p* > 0.05	*p* > 0.05	*p* > 0.05	*p* > 0.05	*p* > 0.05	*p* = 0.010; cv = 0.212
Have you had gastric disease associated with Helicobacter infection?	Yes/No	p > 0.05	*p* > 0.05	*p* > 0.05	*p* > 0.05	*p* = 0.029; cv = 0.179	*p* > 0.05	*p* = 0.037; cv = 0.171	*p* = 0.040; cv = 0.169
Have you had treatment for *Helicobacter* spp. infection?	Yes/No	*p* = 0.003; cv = 0.277	*p* > 0.05	*p* = 0.020; cv = 0.268	*p* > 0.05	*p* > 0.05	*p* > 0.05	*p* = 0.029; cv = 0.219	*p* = 0.23; cv = 0.226
Do you consider hygiene and safety measures during food preparation?	Yes/No	*p* > 0.05	*p* = 0.036; cv = 0.172	*p* > 0.05	*p* > 0.05	*p* > 0.05	*p* < 0.01; cv = 0.398	*p*>0.05	*p*>0.05

cv = Cramer’s V.

## Data Availability

Not applicable.
